# ﻿*Somnuekiaflaviflora* (Malvaceae, Brownlowioideae), a new genus and species from Thailand

**DOI:** 10.3897/phytokeys.254.141219

**Published:** 2025-04-03

**Authors:** Porntawat Chalermwong, Sutee Duangjai, Aroon Sinbumroong, Theerawat Thananthaisong, Kunanon Daonurai, Anusara Kaewmuan, Manop Poopath, Wanwisa Bhuchaisri, Kusol Tangjaipitak, Bhanumas Chantarasuwan, Chatchai Ngernsaengsaruay, Sukid Rueangruea, Somran Suddee

**Affiliations:** 1 Protected Area Regional Office 4 (Surat Thani), Department of National Parks, Wildlife and Plant Conservation, Surat Thani, 84000, Thailand; 2 Department of Forest Biology, Faculty of Forestry, Kasetsart University, Bangkok, 10900, Thailand; 3 Surat Thani National Park and Protected Area Innovation Center, National Park Innovation Institute, Department of National Parks, Wildlife and Plant Conservation, Surat Thani, 84000, Thailand; 4 Forest Herbarium (BKF), Department of National Parks, Wildlife and Plant Conservation, Bangkok, 10900, Thailand; 5 National Science Museum, Thailand, Khlong 5, Klong Luang, Pathumthani, 12120, Thailand; 6 Department of Botany, Faculty of Science, Kasetsart University, Bangkok, 10900, Thailand; † Deceased

**Keywords:** Endemic, monotypic, phylogenetic, taxonomy

## Abstract

*Somnuekia* is described as a new genus of Malvaceae, currently known only from a few locations in the upper part of Tenasserim in northern and peninsular Thailand. Morphological and molecular phylogenetic analyses, based on plastid DNA sequence regions, support the recognition of this new genus within the Brownlowioideae (Malvaceae). Its distinct phylogenetic position, along with a distinct set of morphological and palynological characteristics, strongly support the recognition of *Somnuekia* as a new genus. A formal description of *Somnuekiaflaviflora* is provided along with illustrations, photographs, a distribution map and conservation notes. Furthermore, this new genus is compared to other Asian genera within the subfamily.

## ﻿Introduction

In July 2018, Porntawat Chalermwong and his colleague discovered an unknown Malvaceae species along the trail to Dad Fa Waterfall in Tai Rom Yen National Park, Surat Thani Province, peninsular Thailand. In January 2019, they conducted a follow-up survey of the area to collect flowering and immature fruiting specimens. Several characteristics indicated that this species belonged to the expanded family Malvaceae Juss. (Bayer and Kubitzki 2003), including its tree habit, simple leaves, stellate indumentum, 5-locule capsule opening loculicidally and subglobose seeds. The leaves of this species resemble those of *Pityranthetrichosperma* (Merr.) Kubitzki, a member of the Brownlowioideae Burrett from China, but its fruit is distinct. This species has a loculicidal capsule, 4.9–6.0 cm long, with five locules, each containing 6–8 seeds (vs. 2–2.5 cm long, with each locule containing 1–2 seeds). Consequently, the unknown species was initially thought to belong to the Brownlowioideae. Leaf samples were then sent to Sutee Duangjai for clarification of the subfamily placement using *ndhF* DNA sequence data. Preliminary results confirmed its placement within the Brownlowioideae. Subsequently, flowering and immature fruiting specimens of the unknown Malvaceae were sent to the Faculty of Forestry at Kasetsart University for classical taxonomic investigation. The samples could not be identified using the key to genera of Brownlowioideae (Bayer and Kubitzki 2003), nor did they match any known genus of the subfamily (Bayer and Kubitzki 2003; Cheek 2007). Therefore, it was considered to represent a new taxon.

The Brownlowioideae, one of ten subfamilies of Malvaceae, consists of nine genera (Colli-Silva et al. 2025) and approximately 97 species (POWO 2025), primarily found in the Old World (Bayer and Kubitzki 2003; Nyffeler et al. 2005). Members of Brownlowioideae are distinguished by stamens with thecae that are divergent at the base, but convergent at the top of the connective (Burret 1926; Bayer et al. 1999; Nyffeler et al. 2005) and sepals fused to form a persistent, campanulate or urceolate calyx (Nyffeler et al. 2005; Cvetković et al. 2021). Colli-Silva et al. (2025) recognised nine genera in the subfamily Brownlowioideae: *Berrya* Roxb., *Brownlowia* Roxb., *Carpodiptera* Griseb., *Christiana* DC., *Diplodiscus* Turcz., *Indagator* Halford, *Jarandersonia* Kosterm., *Pentace* Hassk. and *Pityranthe* Thwaites. The largest genus is *Pentace* (ca. 30 spp.), followed by *Brownlowia* (ca. 29 spp.) and *Diplodiscus* (11 spp.). The remaining genera each contain fewer than 10 species: *Jarandersonia* (eight spp.), *Berrya* (six spp.), *Christiana* (six spp.), *Carpodiptera* (four spp.), *Pityranthe* (two spp.) and *Indagator* (one sp.) (POWO 2025).

Six genera of the subfamily Brownlowioideae are distributed across Asia, New Guinea and the Pacific Islands (Bayer and Kubitzki 2003): *Berrya*, *Brownlowia*, *Diplodiscus*, *Jarandersonia*, *Pentace* and *Pityranthe*. *Indagator*, a monotypic genus endemic to Australia, was placed close to *Diplodiscus*, *Jarandersonia* and *Pityranthe* by Cheek (2007), based on fruit characteristics. The other two genera, *Carpodiptera* and *Christiana*, have a disjunct distribution in Africa and the Americas (Bayer and Kubitzki 2003; Renner 2004) and are genetically close to *Berrya* (Hernández-Gutiérrez and Magallón 2019; Barbosa-Silva et al. 2021). Only three genera of the Brownlowioideae have been reported in Thailand (Phengklai 1993, as “Tiliaceae”): *Berrya*, *Brownlowia* and *Pentace*, with a total of approximately eight species. In China, one species each of *Berrya* and *Pityranthe* (as *Hainaniatrichosperma* Merr. or *Diplodiscustrichosperma* (Merr.) Y. Tang, M.G. Gilbert & Dorr) has been reported (Hung-Ta and Ru-Huai 1989; Bayer and Kubitzki 2003; Tang et al. 2007). In Burma (now Myanmar), only three genera, *Berrya*, *Brownlowia* and *Pentace*, have been recorded (Kurz 1877), with approximately five species. These same genera have also been reported in Indo-China (Gagnepain 1910, 1945). In Malaysia, the subfamily consists of five genera — *Berrya*, *Brownlowia*, *Diplodiscus*, *Jarandersonia* and *Pentace* — and includes about 55 species (Tan et al. 2011).

The development of molecular phylogenetic approaches has expanded our understanding of the phylogeny and systematics of Malvaceae*sensu lato* (Alverson et al. 1999; Bayer et al. 1999; Nyffeler and Baum 2000, 2001; Whitlock et al. 2001; Nyffeler et al. 2005; Wilkie et al. 2006; Le Péchon et al. 2010, 2015; Brunken and Muellner 2012; Richardson et al. 2015; Areces-Berazain and Ackerman 2016; Carvalho-Sobrinho et al. 2016; Hernández-Gutiérrez and Magallón 2019; Dorr and Wurdack 2021; Wu et al. 2023; Hanes et al. 2024; Zhong et al. 2024; Colli-Silva et al. 2025). However, amongst the subfamilies within Malvaceae, only Brownlowioideae remains poorly characterised phylogenetically. The most recent and comprehensive phylogenetic study of Brownlowioideae, conducted by Hernández-Gutiérrez and Magallón (2019), included seven of the 95 species in the subfamily, each representing a different genus. Their results divided the subfamily into two clades. The first clade comprises *Brownlowiaelata* Roxb., *Diplodiscuspaniculatus* Turcz., *Jarandersoniaclemensiae* (Burret) Kosterm. and *Pentacepolyantha* Hassk. The second clade consists of *Berryajavanica* (Turcz.) Burret, *Carpodipteracubensis* Griseb. (*Ca.ameliae* Lundell) and *Christianaafricana* DC. Due to the lack of phylogenetic studies, generic circumscription within Brownlowioideae has not yet been confirmed using phylogenetic data. Currently, only three complete chloroplast genomes of *Pi.trichosperma* and 51 plastid DNA sequence accessions for members of Brownlowioideae are available in GenBank (https://www.ncbi.nlm.nih.gov/; accessed April 2024). All prior phylogenetic analyses of the subfamily Brownlowioideae have been based on plastid DNA sequences, such as *atpB*, *ndhF*, *matK* and *rbcL* (Alverson et al. 1999; Bayer et al. 1999; Nyffeler et al. 2005; Hernández-Gutiérrez and Magallón 2019). Amongst these regions, *ndhF* has been commonly used as a marker in studies on the phylogenetic relationships within Malvaceae*s.l.* (Alverson et al. 1999; Whitlock et al. 2001; Pfeil et al. 2002; Nyffeler et al. 2005; Wilkie et al. 2006; Koopman and Baum 2008; Richardson et al. 2015; Hernández-Gutiérrez and Magallón 2019). In this study, we reconstructed a phylogenetic tree of Malvaceae*s.l.* to determine the subfamily placement of an unknown Thai Malvaceae species. We also evaluated phylogenetic relationships with other genera within the subfamily using DNA sequences from three plastid regions: *ndhF*, *rbcL* and the *trnL* intron and the *trnL-trnF* spacer (hereafter the *trnLF* region).

**Figure 1.**
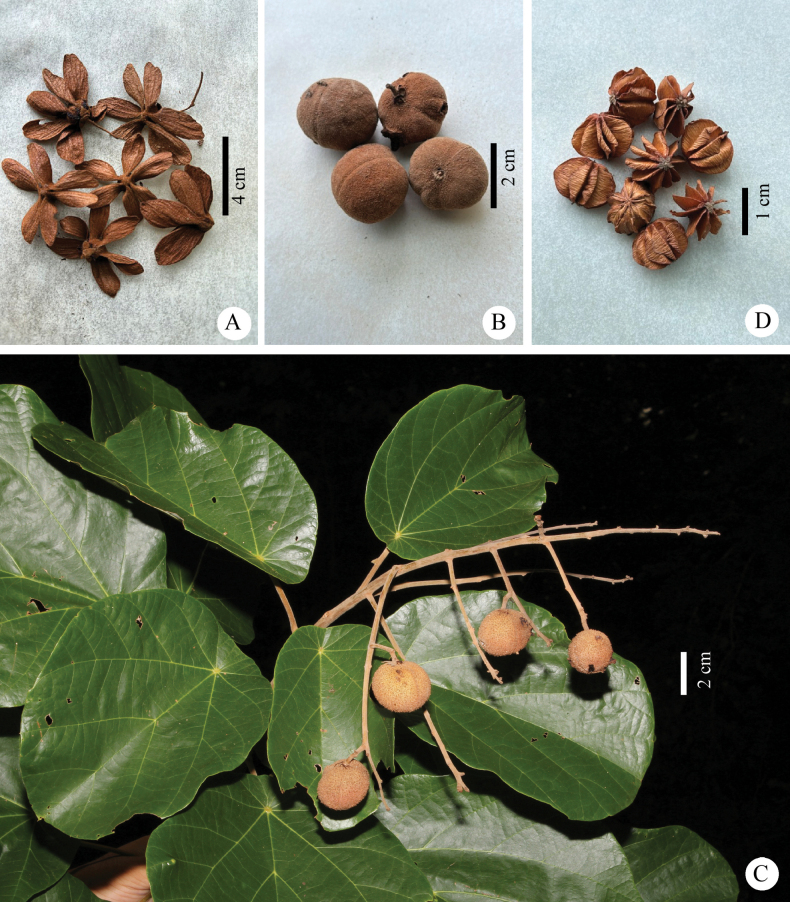
Fruit morphology of some Asian genera of the Brownlowioideae**A***Berrya* (*D. Praphat 124*, BKF) **B, C***Brownlowia* (**B***S. Pinnin 1548*, BKF **C** living plant) **D***Pentace* (*C. Niyomdham & P. Puudjaa 7500*, BKF).

All genera in the Brownlowioideae are delimited, based on fruit characters considered taxonomically essential (Kurz 1877; Kostermans 1960, 1961a, 1961b, 1964, 1970; Kochummen 1973; Hung-Ta and Ru-Huai 1989; Phengklai 1993; Bayer and Kubitzki 2003; Cheek 2007; Tang et al. 2007; Tan et al. 2011; Chung and Soepadmo 2017; Ganesan et al. 2021; Coutinho et al. 2025), whereas floral characters, except for staminode presence, are not useful at the generic level (Kostermans 1960, 1961a, 1961b, 1964, 1970; Cheek 2007). The genus *Brownlowia* can be distinguished from other related genera — *Berrya*, *Carpodiptera*, *Christiana*, *Diplodiscus*, *Indagator*, *Jarandersonia*, *Pentace* and *Pityranthe* — by its apocarpous and loosely connected carpels and indehiscent fruits (Fig. 1). In contrast, the genera *Berrya*, *Carpodiptera*, *Christiana*, *Diplodiscus*, *Indagator*, *Jarandersonia*, *Pentace* and *Pityranthe* possess united carpels. The genera *Berrya* and *Pentace* have winged capsules (Fig. 1), but the latter also has staminodes. *Diplodiscus* has exalate capsules, whereas *Pityranthe* has thin woody capsules. *Jarandersonia* has spinulose fruits similar to those of *Indagator*, which has a woody capsule with a shorter spine and both lack wings. Most of the Asian genera, except *Berrya*, possess five staminodes. The genera *Carpodiptera* and *Christiana* have only fertile stamens; their fruits are winged and woody capsules, respectively.

The palynological features of most genera within Brownlowioideae were described by Perveen et al. (2004), who conducted light microscopy (LM) and scanning electron microscopy (SEM) for eight genera (*Berrya*, *Brownlowia*, *Carpodiptera*, *Christiana*, *Diplodiscus*, *Jarandersonia*, *Pentace*, and *Pityranthe* [including *Hainania* Merr.]). The pollen grains of these eight genera are shed as monads with tricolporate apertures (short colpi), suboblate or oblate in shape and typically with a reticulate tectum (Perveen et al. 2004).

To evaluate the taxonomic status of the unknown Thai Malvaceae species, molecular phylogenetic studies were performed to determine its genus and subfamily placement. Additionally, the morphological characteristics of the new taxon were assessed. Based on the results of phylogenetic and morphological analyses, we describe this plant as a new species, *Somnuekiaflaviflora*, in a monotypic new genus, *Somnuekia*, belonging to the subfamily Brownlowioideae.

## ﻿Materials and methods

### ﻿New taxon sampling, morphological investigation, description and geographical distribution

The first set of specimens of the new species was collected by Aroon Sinbumroong and Porntawat Chalermwong in Surat Thani Province, Peninsular Thailand, between January 2019 and January 2023. The second and third sets were collected in Tak Province, northern Thailand, during February and November 2022 by Somran Suddee and his team from BKF. The fourth set, collected by Manop Poopath in Kamphaeng Phet Province, northern Thailand, in 2015, was located in the BKF. Most specimens were preserved in alcohol and pressed and dried at the office in Bangkok. Vouchers were deposited at the Bangkok Herbarium (BK), BKF and the Royal Botanic Gardens, Kew (K) (abbreviations follow Thiers 2020). The collected material was photographed in the field and at the Department of Forest Biology, Faculty of Forestry, Kasetsart University. Morphological studies of the new species were based on observations of living plants, field notes, photographs and dried herbarium specimens. Trichomes on branches, petioles, leaf blades, fruits and seeds were examined from fresh, alcohol-preserved and dried samples using a Zeiss Stemi 508 apochromatic compact stereomicroscope (Carl Zeiss, Germany) and a Zeiss Axioskop 40 microscope (Carl Zeiss, Germany) at the Department of Forest Biology, Faculty of Forestry, Kasetsart University. Images were captured using a Canon EOS700D digital camera and processed with AxioVision SE64 software (Carl Zeiss). The stamen characters and sequential maturation were observed on both fresh and preserved flowers. Pollen samples were taken from a herbarium specimen collected from Surat Thani Province (Chalermwong & Sinbumroong 20230103-01). Pollen morphological characters were observed using LM and SEM at the Department of Botany and the Scientific Equipment Center, Faculty of Science, Kasetsart University. For LM, pollen grains (20 tetrads, 60 grains) were examined with a Zeiss Axioskop 40 microscope (Carl Zeiss, Germany) and images were captured using a Canon EOS700D digital camera. Pollen grains were mounted on stubs with double-sided sellotape, sputter-coated with gold and examined using an FEI Quanta 450 SEM (Hillsboro, OR, USA) at 15.00 kV. Pollen morphology was described following Erdtman (1952). The characters of the unknown Malvaceae taxon were compared with those of six Asian genera within the subfamily Brownlowioideae to clarify morphological similarities and differences.

Digital images of type specimens of other genera in the subfamily Brownlowioideae, available from JSTOR Global Plants (http://plants.jstor.org/), the Herbarium Catalogue, Royal Botanic Gardens, Kew (http://www.kew.org/herbcat), the BioPortal of Naturalis Biodiversity Center (http://bioportal.naturalis.nl/), the Museum National d’Histoire Naturelle-Paris Herbarium, P (https://science.mnhn.fr/institution/mnhn/collection/p/) and the PE Herbarium (https://petype.myspecies.info), as well as collections in BK and BKF, were examined and compared with the new species.

Relevant taxonomic studies (e.g. Kurz (1877); Gagnepain (1910, 1945); Kostermans (1960, 1961a, 1961b, 1964, 1970); Kochummen (1973); Hung-Ta and Ru-Huai (1989); Phengklai (1993); Bayer and Kubitzki (2003); Cheek (2007); Tang et al. (2007); LaFrankie (2010); Tan et al. (2011)) were consulted.

A distribution map based on specimens and field observations was created using ArcMap version 10.3.1 (ESRI). The conservation status of the species was assessed by calculating its extent of occurrence (EOO) and area of occupancy (AOO) using GeoCAT, then evaluated according to the IUCN Red List categories and criteria (IUCN 2022).

### ﻿Molecular phylogenetic analysis

#### ﻿Sampling, DNA markers, DNA extraction, PCR amplification and sequencing

Since the preliminary results, based on the *ndhF* gene, indicate that the unknown taxon is a member of the Brownlowioideae, then sixteen species (21 samples) of Brownlowioideae, representing nine genera, were analysed. Only the Australian monotypic genus *Indagator* was not included. Thirty-five new sequences were generated for this study, sequences of other Brownlowioideae members were obtained from previous studies. Additional sequences from the complete chloroplast genomes of 21 taxa representing the other eight Malvaceae subfamilies (except only the member of the Matisioideae) were downloaded from GenBank. *Muntingiacalabura* L. was used as an outgroup. We used DNA sequences from three plastid regions (*ndhF*, *rbcL* and the *trnLF* region) to investigate the subfamily placement of the unknown Malvaceae taxon from Thailand and its relationships with other genera within the subfamily. Taxon names and GenBank accession numbers are available in Appendix 1.

Leaves from the unknown species and nine other samples were dried and preserved in silica gel (Chase and Hills 1991). Total DNA was extracted from silica-dried leaf samples using either a modified 2 × cetyltrimethyl ammonium bromide (CTAB) procedure (Doyle and Doyle 1987) or the DNeasy Plant Mini Kit (Qiagen, Valencia, CA, USA). DNA quality and quantity were assessed by agarose gel electrophoresis. Double-stranded DNA from three chloroplast genome regions was amplified by polymerase chain reaction (PCR) using six primers for *ndhF*, four for *rbcL* and two for the *trnL* intron and *trnL-F* spacer region (Table 1). PCR was performed on a C1000 Thermal Cycler (Bio-Rad, Singapore) in a volume of 50 μl, containing 25 μl of 2 × DreamTaq Green PCR Master Mix (Thermo Fisher Scientific, Waltham, MA, USA), 21 μl of nuclease-free water, 1 μl of bovine serum albumin (BSA) (New England Biolabs), 1 μl of each primer (20 mmol/l) and 1 μl of template DNA. The thermocycler protocol consisted of an initial 3-min pre-melt at 94 °C, followed by 35 cycles (denaturation for 1 min at 94 °C, annealing for 1 min at 50 °C and extension for 1 min at 65 °C), with a final extension for 10 min at 65 °C.

**Table 1.** Primers used for DNA amplification and sequencing of the three plastid regions.

Region and Primer name	Sequence (5′ → 3′)	Forward or reverse primer	Source
*ndhF* gene			
40F	ATATTCATGGATCATACCTTTTGTG	forward	This study
1080R	TAAAAGGAATGCTGTAAATATTCCG	reverse	This study
972F	GTCCCAACTGGGTTATATGATG	forward	Alverson et al. (1999)
1860R	TAAAAGGAATGCTGTAAATATTCCG	reverse	This study
1300F	GTGACAGTTGGTTGTATTCACCGA	forward	This study
2140R	TCTTATACCTTTTGTTAAGGATAT	reverse	This study
*rbcL* gene			
rbcL1F	ATGTCACCACAAACAGAAAC	forward	Savolainen et al. (2000)
rbcL724R	TCGCATGTACCTGCAGTAGC	reverse	Fay et al. (1997)
rbcL636F	TGCGTTGGAGAGACCGTTTC	forward	Kaufmann and Wink (1994)
rbcL1R	TCCTTTTAGTAAAAGATTGGGCCGAG	reverse	Savolainen et al. (2000)
*trnL* intron and *trnL*-*F* spacer			
c	CGAAATCGGTAGACGCTACG	forward	Taberlet et al. (1991)
f	ATTTGAACTGGTGACACGAG	reverse	Taberlet et al. (1991)

Amplified products were cleaned using FastAP Thermosensitive Alkaline Phosphatase and Exonuclease I (Thermo Fisher Scientific). The cleaned PCR products were sequenced using the same primers as in the initial amplifications. Sanger sequencing was performed at the Macrogen Sequencing Facility (Macrogen, Inc., Seoul, South Korea).

#### ﻿Sequence editing, alignment and phylogenetic analysis

Raw sequences were edited and assembled using AutoAssembler version 1.4.0 (Applied Biosystems). Multiple-sequence alignments were performed with ClustalX (Larkin et al. 2007) and manually adjusted in MacClade 4.07 (Maddison and Maddison 2005). Phylogenetic analyses were conducted using maximum parsimony (MP) and Bayesian Inference (BI; Rannala and Yang (1996); Yang and Rannala (1997)). MP analyses were performed using equally weighted, unordered nucleotide substitutions (Fitch 1971) in PAUP* version 4.0b10 (Swofford 2002). The most parsimonious trees were identified through heuristic searches with 1,000 replicates of random sequence addition, using tree bisection and reconnection (TBR) swapping and the setting MulTrees = on. TBR swapping was applied to a maximum of 200 trees (nchuck = 200) per replicate. Node support was assessed using bootstrap with 1,000 replicates, the heuristic search with simple addition sequences, TBR swapping (nchuck = 200) and setting MulTrees = off. BI was conducted using MrBayes version 3.2 (Ronquist et al. 2012). Nucleotide substitution models were selected, based on Akaike’s Information Criterion (AIC), implemented in MrModelTest version 2.3 (Nylander 2004). Two independent Markov Chain Monte Carlo analyses were performed, each with four simultaneous chains over 10,000,000 generations, sampling one tree every 1,000 generations. The first 25% of trees were discarded as burn-in and the remaining trees were used to construct a majority-rule consensus tree with Bayesian posterior probabilities (PPs).

## ﻿Results and discussion

### ﻿Phylogenetic placement of the unknown taxon within Brownlowioideae and its relationships with other genera based on three plastid regions

**Figure 2.**
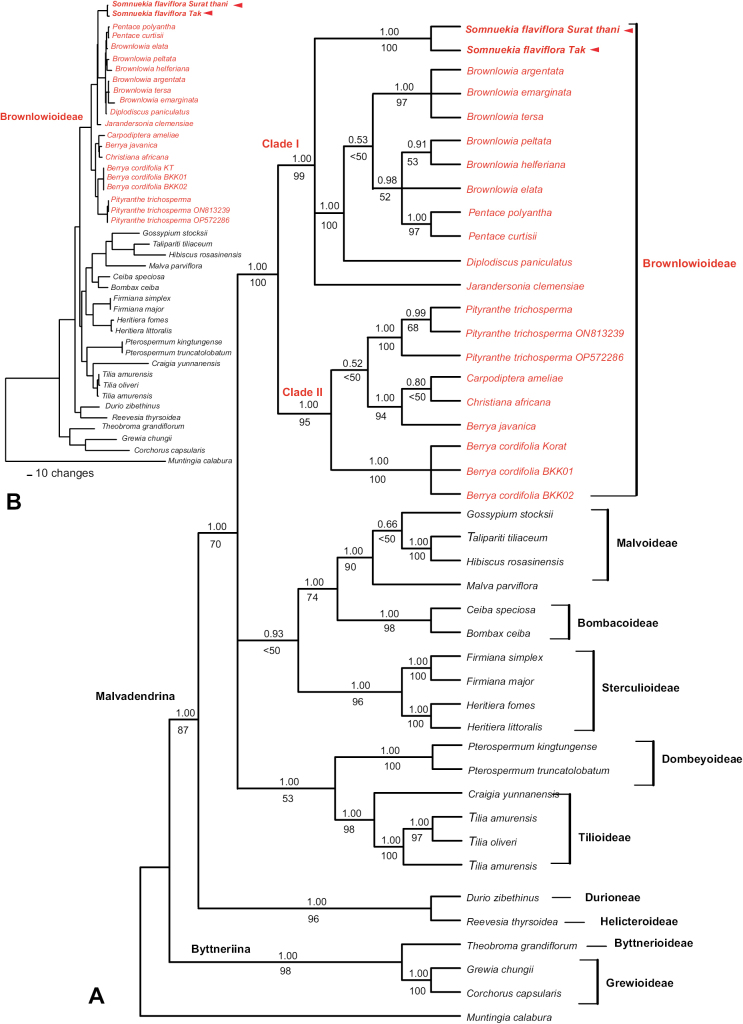
Phylogenetic trees of the subfamily Brownlowioideae resulting from analysis of the concatenated dataset (*ndhF* + *rbcL* + *trnLF* region) of 43 samples **A** fifty percent Bayesian majority-rule consensus tree. Posterior probability values are shown above branches and bootstrap percentages for supported clades are shown below branches. Individuals of *Somnuekiaflaviflora* are in bold and indicated by arrows. Subfamilies *sensu*Colli-Silva et al. (2025) are labelled where applicable **B** phylogram obtained in Bayesian analysis

Phylogenetic placement of the unknown taxon and its relationships with other genera within the subfamily Brownlowioideae were evaluated by analyzing *ndhF*, *rbcL* and the *trnLF* region. The alignment of this dataset consisted of 4,931 characters, with 1,238 variable sites and 495 parsimony informative sites. The MP heuristic search retrieved the 324 most parsimonious trees, with 1,788 steps (consistency index = 0.80; retention index = 0.76). The best-fitting model of nucleotide evolution was GTR + I + G for all regions. Tree topologies generated from the combined data using BI were approximately congruent with those from MP; only the BI majority tree is shown in Fig. 2. In Malvaceae*s.l.*, all nine subfamilies were monophyletic, consistent with previous reports (Alverson et al. 1999; Nyffeler et al. 2005; Hernández-Gutiérrez and Magallón 2019). There were two main clades within Malvaceae*s.l.*: Byttneriina (PP = 1.0, BS = 98) and Malvadendrina (PP = 1.0, BS = 87). The monophyly of the subfamily Brownlowioideae was strongly supported (PP = 1.0, BS = 100) based on the combined dataset; however, our results do not provide additional insight into the interrelationships beyond those previously published (Alverson et al. 1999; Nyffeler et al. 2005). The sister lineage of Brownlowioideae was not resolved and the subfamily formed a polytomy with the clades Dombeyoideae and Tilioideae (PP = 1.0, BS = 53) and the Bombacoideae/Malvoideae/Sterculioideae clade (PP = 1.0, BS < 50). Phylogenetic analysis of the sequence data of *ndhF*, *rbcL* and the *trnLF* region revealed that the unknown Malvaceae species is a member of Brownlowioideae and confirmed the placement of seven other species: *Berryacordifolia* (Willd.) Burret, *Brownlowiaargentata* Kurz, *Brownlowiaemarginata* Pierre, *Brownlowiahelferiana* Pierre, *Brownlowiapeltata* Benth., *Brownlowiatersa* (L.) Kosterm. and *Pentacecurtisii* King within this subfamily (Fig. 2).

Brownlowioideae contained two clades, Clade I (PP = 1.0, BS = 99) and Clade II (PP = 1.0, BS = 95) (Fig. 2), consistent with the results based on *ndhF* (unpublished data). The first clade included *Br.argentata*, *Br.elata*, *Br.emarginata*, *Br.helferiana*, *Br.peltata*, *Br.tersa*, *D.paniculatus*, *J.clemensiae*, *Pe.curtisii*, *Pe.polyantha* and the unknown Malvaceae. The second clade consisted of *Be.cordifolia*, *Be.javanica*, *Ca.cubensis*, *Ch.africana* and *Pi.trichosperma*. The two accessions of the unknown Malvaceae (*Somnuekiaflaviflora*) were monophyletic with strong support (PP = 1.0, BS = 100).

In Brownlowioideae Clade I, *J.clemensiae* and the unknown taxon (*Somnuekiaflaviflora*) were isolated, while the other samples of the genera *Brownlowia*, *Diplodiscus* and *Pentace* formed a subclade, with *D.paniculatus* as the sister taxon. Therefore, the monophyly of the genus *Brownlowia* was not supported. *Br.argentata*, *Br.emarginata* and *Br.tersa* grouped with high support (PP = 1.0, BS = 97). In contrast, *Br.elata*, *Br.helferiana* and *Br.peltata* formed a clade with two species of *Pentace* (PP = 0.98, BS = 52). The second clade of Brownlowioideae consisted of two species of *Berrya*, *Ca.cubensis*, *Ch.africana* and *Pi.trichosperma* (PP = 1.0, BS = 94). This clade contained three subclades: the first comprised the three samples of *Be.cordifolia*, the type species of the genus (PP = 1.0, BS = 100); the second contained *Be.javanica*, *Ca.cubensis* and *Ch.africana* (PP = 1.0, BS = 94); and the third included all samples of *Pi.trichosperma* (PP = 1.0, BS = 100). The monophyly of the genus *Berrya* was not supported because *Be.javanica* grouped with *Ca.cubensis* and *Ch.africana*, with high support, rather than with the type species *Be.cordifolia*. The results (Fig. 2) indicated that the new species is not closely related to *Pi.trichosperma*, but belongs to the same clade as *Br.argentata*, *Br.elata*, *Br.emarginata*, *Br.helferiana*, *Br.peltata*, *Br.tersa*, *D.paniculatus*, *Pe.curtisii*, *Pe.polyantha* and *J.clemensiae*.

### ﻿Morphological study and taxonomic treatment

After confirming that the collected species belonged to the Brownlowioideae (Malvaceae), we examined all collections of this subfamily from Thailand in BK and BKF. In addition, we reviewed virtual collections of nine genera — *Berrya*, *Brownlowia*, *Carpodiptera*, *Christiana*, *Diplodiscus*, *Indagator*, *Jarandersonia*, *Pentace* and *Pityranthe* — in JSTOR Global Plants, as well as in the K, L, P and PE Herbaria. The collections of the unknown Malvaceae could not be satisfactorily placed in any of the currently described genera within Brownlowioideae. Furthermore, based on the key to genera of Brownlowioideae and descriptions of recognised genera (Bayer and Kubitzki 2003), the unknown Malvaceae is not referable to any recognised genus in the subfamily and is described here as both a new genus and species. The characteristics of the ovaries, fruits and seeds of this new taxon are not consistent with any genera bearing capsular fruits in the subfamily. Morphological characteristics and habitat data of the new taxon, along with those of closely-related Asian genera, are summarised in Table 2.

**Table 2.** Characteristics of the genus *Somnuekia* and closely-related Asian taxa in the subfamily Brownlowioideae.

Character	*Somnuekia*	*Pityranthe*	*Berrya*	*Brownlowia*	*Diplodiscus*	*Pentace*	*Jarandersonia*
Number of species	1	2	6	29	11	30	8
Distribution	Thailand	Sri Lanka and China (Guangxi to Hainan)	Andaman Is., Bangladesh, Borneo, Cambodia, Christmas I., Fiji, India, Java, Laos, Lesser Sunda Is., Malaya, Myanmar, New Guinea, Northern Territory, Philippines, Queensland, Sri Lanka, Sulawesi, Taiwan, Thailand, Vietnam	Andaman Is., Bangladesh, Borneo, Cambodia, India, Laos, Malaya, Maluku, Myanmar, New Guinea, the Philippines, Solomon Is., Sulawesi, Sumatra, Thailand, Vietnam	Borneo, Malaya, the Philippines	Bangladesh, Borneo, Cambodia, Java, Laos, Lesser Sunda Is., Malaya, Myanmar, the Philippines, Sumatra, Thailand	Borneo
Foliaceous staminodes	present, 5	present, 5	absent	present, 5	present, 5	present, 5	present, 5
Pollen grain	tetrahedral tetrads	monads	monads	monads	monads	monads	monads
Carpels	5-loculed, 6–8 ovules per locule, axile placentation	3–5-loculed, 1(2) ovules per locule, axile placentation	3–5-loculed, 2–6 ovules per locule, axile placentation	5-loculed, 2 ovules per locule, axile placentation	5-loculed, 2 ovules per locule, axile placentation	3–5(10)-loculed, 2 ovules per locule, axile placentation	5-loculed, 2 ovules per locule, axile placentation
Fruits	syncarpous fruit has 5-radiating carpels, exalate capsules	syncarpous fruit lacking wing, thinly woody capsules	syncarpous fruit has winged capsules	apocarpous fruit lacking wing	syncarpous fruit lacking wing, exalate capsules	syncarpous fruit has winged capsules	syncarpous fruit covered with spines bearing setose hairs, capsules

**Figure 3.**
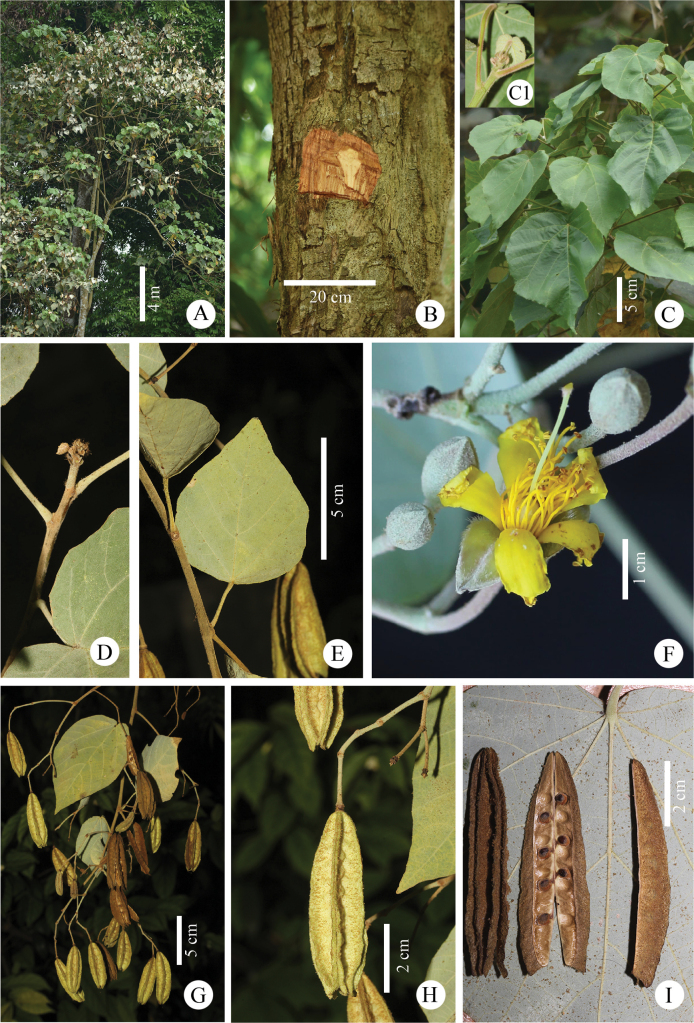
*Somnuekiaflaviflora***A** habit **B** trunk and bark **C** lower-branch shoot and leaves **C1** apical shoot of the lower-branch shoot, showing filiform stipules and trichome covering **D** terminal bud of upper branch shoot showing scale covering **E** leaf-like bracts in infructescence **F** mature flower buds and blooming flower **G** infructescence with immature and mature capsules **H** immature capsules with prominent ridges **I** lateral view of carpel, split longitudinally to show seed arrangement and mature seeds.

**Figure 4.**
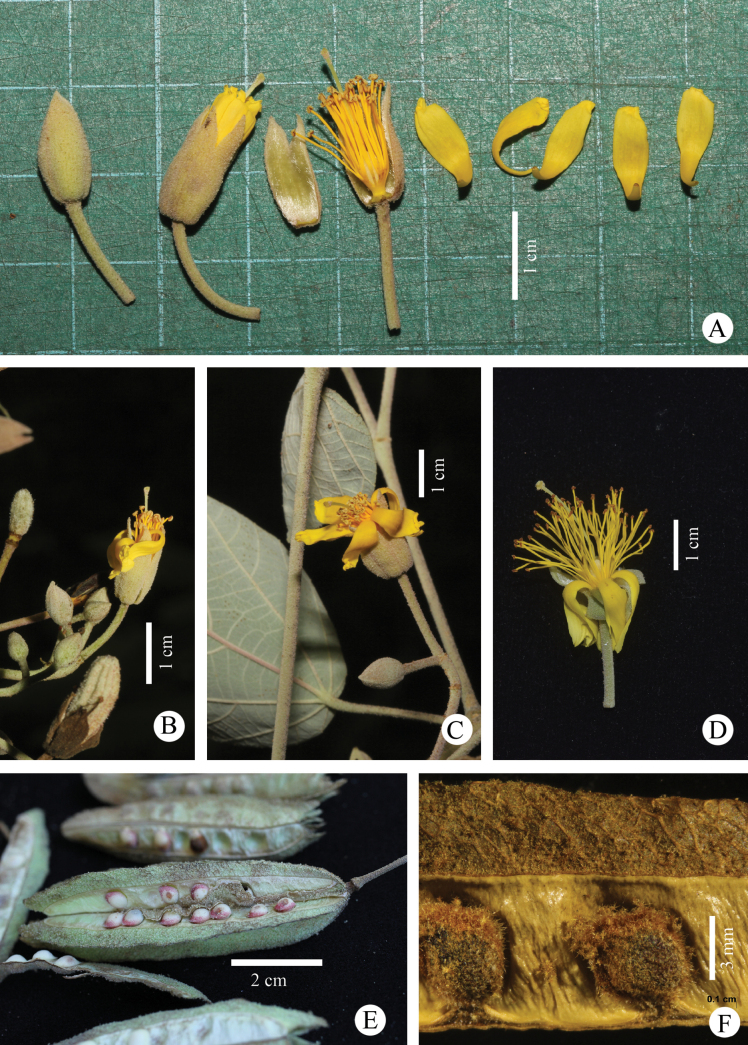
*Somnuekiaflaviflora***A** flowers and flower bud **B–D** flowers at different stages of anthesis **B** early blooming flower with indehiscent anther **C** blooming flower with dehiscent anther **D** late blooming flower with dehiscent anther **E** immature capsules with immature seeds, showing reddish-pink stellate scale covering **F** mature capsule with mature seeds, showing dark brown stellate scale covering. Flowers shown in **A–C** were taken from branches placed in a plastic bag overnight.

**Figure 5.**
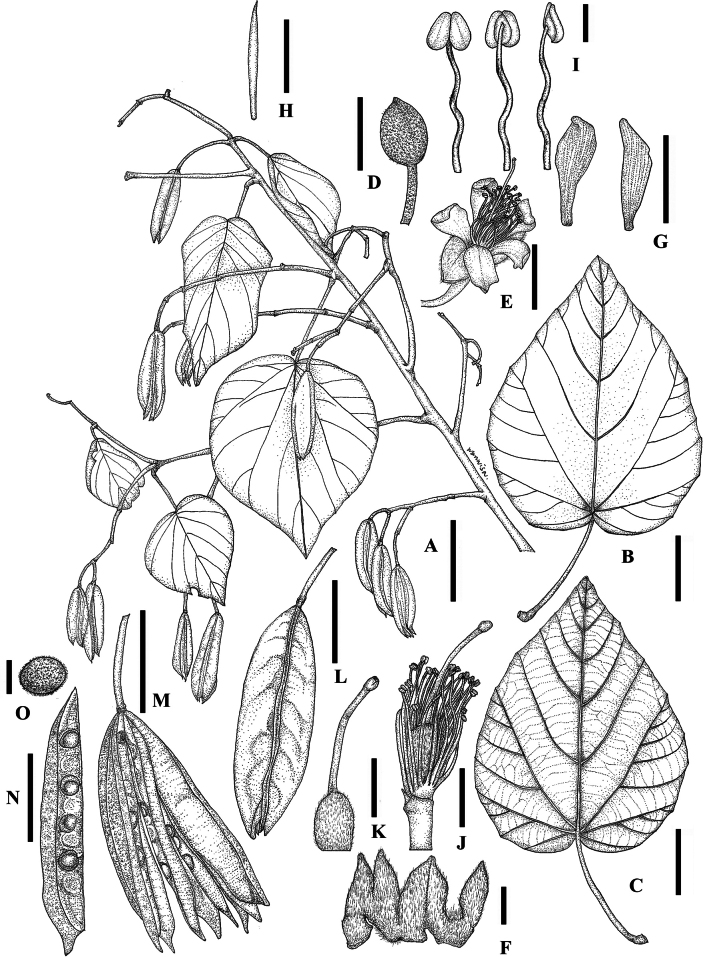
*Somnuekiaflaviflora***A** habit, fruiting branch and leaf-like bracts **B, C** leaves **B** leaf showing adaxial surface **C** leaf showing abaxial surface and venation **D** mature flower bud **E–K** opened flower and its components **E** opened flower **F** sepals showing pilose adaxial surface **G** petals **H** staminode **I** stamens: anterior view (left), posterior view (middle) and lateral view (right) **J** dissected flower with sepals and petals removed, stamens: anterior view (left), posterior view (middle) and lateral view (right) **K** pistil **L** mature capsules **M** mature capsules at splitting stage **N** carpel in lateral view, split longitudinally to show seed arrangement **O** mature seeds. **A–C**, **L–O** from *Chalermwong et al. 20220317-01* (type) **D–K** from *Sinbumroong & Chalermwong 20190128-01*. Drawings by W. Bhuchaisri. Scale bars: 5 cm (**A–C**); 1 cm (**D, E**); 5 mm (**F**); 1 cm (**G**); 5 mm (**H**); 1 mm (**I**); 5 mm (**J**); 5 mm (**K**); 2 cm (**L–N**); 2 mm (**O**).

**Figure 6.**
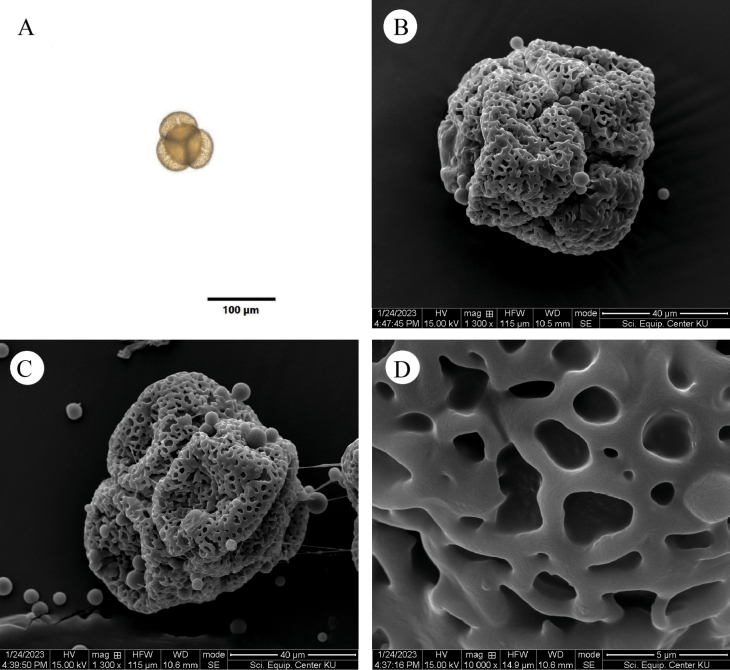
Light and scanning electron micrographs of pollen of *Somnuekiaflaviflora***A** overall view of tetrahedral tetrad (LM, tetrahedral position) **B**–**C** overall view of tetrahedral tetrad and details of exine sculpturing and colpus (SEM, collapsed pollen) **D** detail of exine sculpturing (SEM) showing rugulate-reticulate to reticulate pattern.

The unknown Malvaceae has hermaphroditic flowers with stamens whose thecae are divergent at the base, but convergent at the top of the connective, a characteristic that clearly places it within the subfamily Brownlowioideae. It also has five fusiform staminodes, which are connate at the base with the fertile stamens (Figs 3–5) and differ from those of *Berrya*. The pollen grains of the unknown Malvaceae are grouped in tetrahedral tetrads; however, in SEM-prepared pollen samples, they appeared more or less collapsed (Fig. 6). Only monads with tricolporate (short colpi), suboblate or oblate shapes and typically a reticulate tectum have been reported in the other eight genera (*Berrya*, *Brownlowia*, *Carpodiptera*, *Christiana*, *Diplodiscus*, *Jarandersonia*, *Pentace* and *Pityranthe* [Perveen et al. 2004]). The ovary of the new taxon is ellipsoid in shape and has five carpels, each containing eight ovules with axile placentation.

The unknown Malvaceae has loculicidal dehiscent, cylindrical fruits with five radiating carpels (Figs 3–5), differing from those of *Berrya* (winged capsules vs. wingless capsules), *Brownlowia* (apocarpous vs. syncarpous and indehiscent fruits vs. dehiscent fruits), *Diplodiscus* (two ovules per locule vs. eight ovules per locule), *Jarandersonia* (spinulose fruits vs. non-spinulose fruits), *Pentace* (winged capsules vs. wingless capsules) and *Pityranthe* (five radiating carpels and exalate capsules vs. non-radiating carpels).

### ﻿Taxonomic treatment

Based on the morphological and palynological differences between the undescribed taxon and recognised genera in the subfamily Brownlowioideae, as well as its well-supported, isolated phylogenetic placement (Fig. 2), a new genus is proposed here.

#### ﻿Subfam. Brownlowioideae Burret

##### ﻿ Somnuekia

Taxon classificationPlantaeMalvalesMalvaceae

Duangjai, Chalermw., Sinbumr. & Suddee gen. nov.

5B22AB85-6681-5323-824E-A4045C85F808

urn:lsid:ipni.org:names:77359677-1

###### Type and only known species.

*Somnuekiaflaviflora* Duangjai, Chalermw., Sinbumr. & Suddee

###### Diagnosis.

*Somnuekia* Duangjai, Chalermw., Sinbumr. & Suddee resembles *Pityranthe* Thwaites morphologically, but differs from the latter by its unique pollen, having more numerous ovules in each loculus and fruit characters. The detailed distinguishing characters of this new genus and other genera are listed in Table 2.

###### Description.

Tree, monoecious. Leaves simple, ovate, cordate or slightly five-angled, with 5–7 basal veins, entire with glandular teeth; with long petioles; stipules filiform, caducous. Flowers bisexual, arranged in terminal panicles; bracts slender, caducous; sepals connected into a campanulate tube, calyx teeth 5; petals 5, spathulate; staminodes 5, fusiform; stamens 25–50, filaments slightly connate at base, with elongated filaments, small anthers, uncommissural antherium; ovary superior, 5 cells, 6–8 ovules per cell; slender style; stigma conical. Capsule cylindrical, loculicidal with 5-radiating carpels, (3)6–8 seeds in each loculus, seeds covered with short stellate scales.

###### Etymology.

The genus is named in honour of the Thai dendrologist, Associate Professor Somnuek Pongumphai.

###### Distribution and habitat.

Endemic to Thailand at 200–580 m altitude.

##### ﻿ Somnuekia flaviflora

Taxon classificationPlantaeMalvalesMalvaceae

Duangjai, Chalermw., Sinbumr. & Suddee sp. nov.

501596DB-18DE-50B7-8B39-8C5CC4CE9BC6

urn:lsid:ipni.org:names:77359678-1

###### Type.

Thailand • Surat Thani Province, Tai Rom Yen National Park, Dad Fa Waterfall, 8°51'49.6"N, 99°28'41.7"E, ca. 240 m alt., *P. Chalermwong, A. Sinbumroong & A. Issarapakdee 20220317-01* (holotype: BKF! [SN 268094]; isotypes BK!, BKF! [SN268095], K!, SING!). Figs 3–5.

###### Description.

Tree to 35 m tall, to 80 cm in diameter at breast height. ***Bark*** outer bark greyish-white or brown, smooth and sparsely lenticellate; inner bark light brown, with multiple layers, up to 4.0 cm thick. ***Twigs*** lower branches with petiolar scars, densely covered with reddish-brown pilose hairs, later glabrescent, sparsely lenticellate; upper branches densely covered with short greyish-white stellate hairs, later grabrescent and dark brown. ***Stipules*** base obliquely ovate, concave, ca. 4 × 3 mm, apex cuspidate, ca. 4–5 mm long, caducous, abaxial hirsute, adaxial glabrous. ***Leaves*** spiral, blades ovate, cordate or slightly five-angled, pale green above, greyish-white below, densely covered with stellate scales with scattered reddish-brown pilose hairs along mid-rib and veins below, glabrous or nearly so above, (8.2)10.5–24 × (6.8)9.1–21.5 cm, slightly heart-shaped or truncate at the base, acute or acuminate at the apex, entire or with small teeth and ciliate, with 5–7 basal veins, prominent below, lateral veins 4–6 on each side, tertiary veins scalariform; petiole (3.1)6.8–22.5 cm long, 2.0–5.0 mm in diameter, swollen at either ends, densely covered in reddish-brown pilose hairs for petioles on lower branches or greyish-white stellate scales for petioles of upper branches. ***Inflorescences*** terminal panicles, up to 40 cm long with many lax flowers, peduncles 0.3–0.5 cm in diameter, pedicels, peduncles and rachis covered with greyish-white short stellate scales; bracts and bracteoles small, caducous, with 4–5 leaf-like bracts attached to the rachis. ***Flowers*** bisexual, pedicellate; buds ellipsoid or ovoid, 10–14 mm × 4–6 mm, densely covered in greyish-white stellate scales; pedicels 8–13.5 mm × 2–3 mm, densely covered in stellate scales. Calyx lobes 5, lanceolate, greyish-white, 10–14 mm × 3 mm, valvate, apex acute, densely stellate greyish-white abaxially, densely long silver adaxially. Petals 5, yellow, spathulate, ca. 12 × 3 mm, base gradually tapering, apex emarginate, glabrous on both sides. Androgynophore short, cylindrical, ca. 1 mm long, glabrous. Staminodes 5, yellow, fusiform, ca. 8 mm × 0.5 mm, shorter than filaments of fertile stamens, alternipetalous, glabrous. Fertile stamens 25–50; yellow, antepetalous phalanges, filaments slightly connate at base into 5 fascicles, 10–15 mm long, glabrous; anthers dorsifixed, dithecous, longitudinally dehiscent, 1–1.5 mm long. Ovary ellipsoid, ca. 4 mm × 2.5 mm, densely stellately hairy, carpels 5, united, each carpel with 6–8 ovules, with axile placentation; style 1, ca. 7 mm long, glabrous; stigma conical. ***Infructescences*** up to 40 cm long, densely covered with greyish-white short stellate scales, leaf-like bracts ovate, 6.8–13.1 cm × 5.4–10 cm, petiole 2.5–7.5 cm long, with 8–22 capsules. ***Capsules*** loculicidal, cylindrical, with 5-radiating carpels, 4.9–6.0 cm × 1.5–2.1 cm, with dense stellate scales; stalk 1.4–1.6 cm long, ca. 0.5 mm thick, covered with stellate scales; each carpel flattened, 4.9–6.0 cm × 0.85–1.0 cm, 2–3 mm thick, bulging at seed, each side of pericarp of carpel partially adnate, margins free and covered with stellate scales. ***Seed*** (3)6–8 seeds per lip, subglobose, 2.5 × 3.5 × 2.0 mm, covered with short stellate scales, dark brown.

###### Specimens examined.

Thailand • Surat Thani [Ban Na San District, Tai Rom Yen National Park, nature trail of Dad Fa Waterfall, 8°51'49.6"N, 99°28'41.7"E, 240 m alt., 28 January 2019, fl. and fr., *Sinbumroong & Chalermwong 20190128-01* (**BKF**), • ibid., 17 March 2022, fr., *Chalermwong et al. 20220317-01* (**BKF! BK! K! SING**!), • ibid., 28 April 2022, *Chalermwong & Sinbumroong 20220428-01* (**BKF**), • ibid., 3 January 2023, fl. and fr., *Chalermwong & Sinbumroong 20230103-01* (**BKF**)]; Tak [Phop Pra District, Namtok Pha Charoen National Park, road to Pa Wai Waterfall, 16°33'55"N, 98°48'47"E, 870 m alt., 19 February 2022, fr., *Thananthaisong et al. 690* (**BKF**), • ibid., 9 April 2022, fr., *Kaewmuan et al. 115* (**BKF**)]; Tak [Umphang District, Mae Klong Khi Village, 16°33'54"N, 98°55'6"E, 580 m alt., 6 November 2022, fl. and fr., *Thananthaisong et al. 955* (**BKF**)]; Kamphaeng Phet [Khlong Lan District, Khlong Lan National Park, Khlong Lan Waterfall, 16°07'49.5"N, 99°16'34.4"E, 200 m alt., 8 April 2015, fr., *Poopath et al. 1025* (**BKF**)].

###### Distribution.

**Figure 7.**
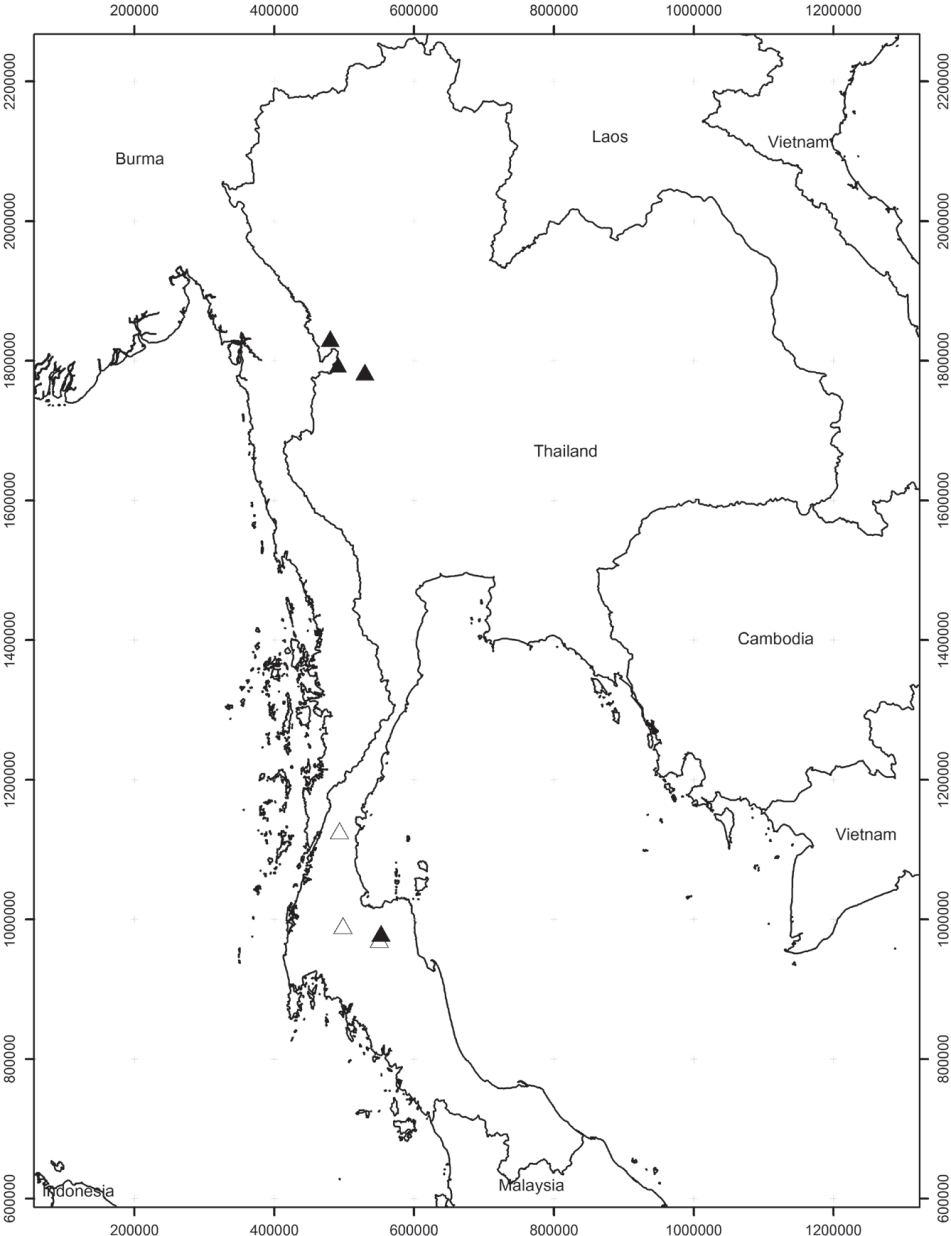
Known distribution of *Somnuekiaflaviflora*. Solid triangles indicate localities of specimen collection; open triangles indicate localities confirmed by field observations by Porntawat Chalermwong and Aroon Sinbumroong. The map was created using ArcMap version 10.3.1 (ESRI).

Endemic. Known from northern and peninsular Thailand. Fig. 7.

###### Palynology.

The pollen grains of this species are grouped in tetrahedral tetrads. The tetrad is 70.78–95.82 (81.46 ± 5.19) µm in diam. Each pollen grain of the tetrad is isopolar and radially symmetrical, circular in polar view. The pollen aperture is tricolporate, the colpus length is 11.78–27.91 (20.13 ± 3.64) µm and the colpus width is 3.15–8.84 (5.69 ± 1.26) µm. The top pollen is 43.69–62.95 (53.17 ± 5.25) µm in diam. The polar axis length of pollen is 40.38–52.25 (44.80 ± 2.65) µm and the equatorial axis width is 48.36–64.46 (55.93 ± 3.19) µm which are large-sized. The shape of pollen is oblate spheroidal or oblate [P/E ratio = 0.71–0.93 (0.80 ± 0.06)]. The exine thickness is 1.92–5.60 (3.88 ± 0.88) µm and the sculpturing is regulate-reticulate or reticulate (Fig. 6).

###### Etymology.

The epithet “flaviflora” refers to the yellow corolla and stamens of the new species.

###### Vernacular name.

The Thai name is “Po Sri Somnuek” (ปอศรีสมนึก).

###### Ecology.

In limestone foothills or the marginal open places of the tropical rain forests and dry evergreen forests; between 200 and 800 m altitude.

###### Conservation status.

According to IUCN (2022) criteria and based on its Area of Occurrence (AOO) of 16 km^2^ and Extent of Occurrence (EOO) of 30,507 km^2^, *Somnuekiaflaviflora* is assessed a preliminary status as Near Threatened B2ab (ii,iii,v). It occurs in seven locations.

###### Phenology.

Flowering November–February, fruiting November–April.

## Supplementary Material

XML Treatment for Somnuekia

XML Treatment for Somnuekia flaviflora

## ﻿Additional information

### Author ORCIDs

Porntawat Chalermwong https://orcid.org/0000-0002-6310-4291

Sutee Duangjai https://orcid.org/0000-0002-1490-759X

Aroon Sinbumroong https://orcid.org/0000-0001-8504-4106

Theerawat Thananthaisong https://orcid.org/0000-0003-0665-2141

Kunanon Daonurai https://orcid.org/0000-0003-4070-032X

Anusara Kaewmuan https://orcid.org/0000-0002-5578-6130

Manop Poopath https://orcid.org/0000-0002-9871-5295

Bhanumas Chantarasuwan https://orcid.org/0000-0002-6106-9875

Chatchai Ngernsaengsaruay https://orcid.org/0000-0002-7131-976X

Sukid Rueangruea https://orcid.org/0000-0003-1512-5976

Somran Suddee https://orcid.org/0000-0003-4031-9721

### Data availability

All of the data that support the findings of this study are available in the main text.

## ﻿Appendix 1

Species names and GenBank accession numbers of the *ndhF*, *rbcL* and *trnLF* sequences used in this study. Newly-generated sequences are in bold and * denotes GenBank accession numbers of the complete chloroplast genomes that were used for analysis.

**Malvaceae**:

**Bombacoideae**: *Bombaxceiba* L., MG569974*, MG569974*, MG569974*; *Ceibaspeciosa* (A.St.-Hil., A.Juss. & Cambess.) Ravenna, MK820674*, MK820674*, MK820674*.

**Brownlowioideae**: *Berryajavanica* (Turcz.) Burret, AF111755, AJ233146, - ; *Berryacordifolia* (Willd.) L.Laurent, **PP750894**, **PP750871**, PP750882; *Berryacordifolia* (Willd.) L.Laurent, **PP750895**, PP750872, PP750883; *Berryacordifolia* (Willd.) L.Laurent, PP750896, PP750873, **PP750884**; *Brownlowiaargentata* Kurz, PP750900, **PP750875**, **PP750887**; *Brownlowiaelata* Roxb., AF111756, AJ233147, PP750886; *Brownlowiaemarginata* Pierre, PP750897, **PP750878**, **PP750890**; *Brownlowiahelferiana* Pierre, **PP750898**, **PP750879**, PP750891; *Brownlowiapeltata* Benth., **PP750901**, **PP750876**, **PP750888**; *Brownlowiatersa* (L.) Kosterm., **PP750902**, PP750877, PP750889; *Carpodipteracubensis* Griseb., AF111757, -, -; *Christianaafricana* DC., PP750903, AJ233149, - ; *Diplodiscuspaniculatus* Turcz., AF230252, -, - ; *Jarandersoniaclemensiae* (Burret) Kosterm., AF230253, -, - ; *Pentacecurtisii* King PP750899, PP750874, PP750885; *Pentacepolyantha* Hassk., AF111758, AJ233156, - ; *Pityranthetrichosperma* (Merr.) Kubitzki, -, AY328195, AY328160; *Pityranthetrichosperma* (Merr.) Kubitzki, ON813239*, ON813239*, ON813239*; *Pityranthetrichosperma* (Merr.) Kubitzki, OP572286*, OP572286*, OP572286*; *Somnuekiaflaviflora* sp. nov., **PP750892**, **PP750869**, PP750880; *Somnuekiaflaviflora* sp. nov., PP750893, **PP750870**, **PP750881**.

**Byttnerioideae**: *Theobromagrandiflorum* (Willd. ex Spreng.) K.Schum., JQ228388*, JQ228388*, JQ228388*.

**Dombeyoideae**: *Pterospermumkingtungense* C.Y.Wu ex H.H.Hsue, MH606238*, MH606238*, MH606238*; *Pterospermumtruncatolobatum* Gagnep., MN533971*, MN533971*, MN533971*.

**Durioneae Becc.**: *Duriozibethinus* L., MG138151*, MG138151*, MG138151*.

**Grewioideae**: *Corchoruscapsularis* L., MK251464*, MK251464*, MK251464*; *Microcoschungii* (Merr.) Chun, MN533967*.

**Helicteroideae s.s.**: *Reevesiathyrsoidea* Lindl., MH939148*, MH939148*, MH939148*.

**Malvoideae**: *Gossypiumstocksii* Mast., JF317355*, JF317355*, JF317355*; *Hibiscusrosa-sinensis* L., MK382984*, MK382984*, MK382984*; *Taliparititiliaceum* (L.) Fryxell, MN826059*, MN826059*, MN826059*; *Malvaparviflora* L., MK860036*, MK860036*, MK860036*.

**Sterculioideae**: *Firmianamajor* (W.W.Sm.) Hand.-Mazz., MG229069*, MG229069*, MG229069*; *Firmianasimplex* (L.) W.Wight, MH671308*, MH671308*, MH671308*; *Heritierafomes* Banks, MK033519*, MK033519*, MK033519*; *Heritieralittoralis* Aiton, MK033518*, MK033518*, MK033518*.

**Tilioideae**: *Craigiayunnanensis* W.W.Sm. & W.E.Evans, MN088379*, MN088379*, MN088379*; *Tiliaamurensis* Rupr., KT894772*, KT894772*, KT894772*; *Tiliaamurensis* Rupr., MH169579*, MH169579*, MH169579*; *Tiliaoliveri* Szyszyl., KT894774*, KT894774*, KT894774*.

**Muntingiaceae**: *Muntingiacalabura* L., MW038825*, MW038825*, MW038825*.
